# Transcatheter Closure of Perimembranous and Intracristal Ventricular Septal Defects Using Amplatzer Duct Occluder II in Children

**DOI:** 10.1155/2021/4091888

**Published:** 2021-09-11

**Authors:** Shenrong Liu, Wenqian Zhang, Junjie Li, Shushui Wang, Mingyang Qian, Jijun Shi, Yumei Xie, Zhiwei Zhang

**Affiliations:** ^1^Department of Cardiac Pediatrics, Guangdong Cardiovascular Institute, Guangdong Academy of Medical Sciences, Guangdong Provincial People's Hospital, Guangzhou 510080, China; ^2^Department of Cardiovascular Surgery, Guangdong Provincial Hospital of Chinese Medicine, The Second Affiliated Hospital of Guangzhou University of Chinese Medicine, Guangzhou 510120, Guangdong, China

## Abstract

**Background:**

Transcatheter closure of aneurysmal perimembranous ventricular septal defect (pmVSD), pmVSD near the aortic valve, and intracristal VSD (icVSD) with symmetrical or asymmetrical ventricular septal defect occluders still presents significant challenges. We report our experience with transcatheter closure of pmVSD and icVSD using Amplatzer duct occluder II (ADO II) in children.

**Method:**

We retrospectively analyzed all children, who presented to our hospital consecutively between March 2014 and June 2020 for attempted transcatheter closure of pmVSD or icVSD with the ADO II device. Standard safety and last-follow-up outcomes were assessed and compared.

**Results:**

In total, 41 patients underwent transcatheter closure of VSD with the ADO II (28 in pmVSD and 13 in icVSD groups) with a median age of 3.5 years (total range: 0.9 to 12 years) and median weight of 15.0 kg (total range: 10.0 to 43.0 kg). Implantation was successful in 40/41 patients (97.5%, 27/28 in pmVSD group, 13/13 in icVSD group). One patient with mild aortic valve prolapse in pmVSD group developed new-onset moderate aortic regurgitation after a 4/4 mm ADO II was deployed; however, this resolved after the device was retrieved and successfully replaced with a 5 mm zero eccentric VSD occluder. There was no procedure-related mortality. After a median follow-up of six months (total range: 6 to 72 months), complete closure rates were 85.1% and 76.9% among pmVSD and icVSD groups, respectively. In the pmVSD group, one case of new-onset moderate tricuspid regurgitation was observed at six months, and there was one case of severe tricuspid regurgitation that had progressed from mild tricuspid regurgitation at 12 months. No serious complications were noted in the icVSD group.

**Conclusion:**

ADO II provides a safe and reproducible alternative for the closure of perimembranous and intracristal ventricular septal defects with a diameter less than 5 mm in young children.

## 1. Introduction

Perimembranous VSD (pmVSD) accounts for 70% of VSDs, by far the most common form of congenital heart disease [1]. Since the first report of transcatheter VSD closure in 1988 [2], along with the development of closure device (either symmetrical or asymmetrical double disc design), transcatheter closure of pmVSD has become an accepted alternative to open heart surgery in selected cases [3, 4]. However, transcatheter closure of pmVSD associated with aneurysmal tissue or in close proximity to the aortic and tricuspid valves still presents significant challenges [5].

Intracristal VSD (icVSD) accounts for 5–29% of VSDs and was previously considered unsuitable for transcatheter closure because of their proximity to the aortic and pulmonary valves [6]. Zero eccentric occluders have been used to close icVSD, achieving a successful closure rate of >90% [7].However, the incidence of aortic regurgitation requiring surgical repair remains relatively high [7].

Various devices have been used to minimize procedural risks and tackle complicated cases associated with VSD closure [8–10]. For example, the KONAR-MF™ VSD occluder (LifeTech, Shenzhen, China) is designed to provide high conformability to septal defects with a lower risk of heart block and valvular interferences [8]. Patent ductus arteriosus occluders have also been used in pmVSD with the anatomic resemblance to a PDA [9]. However, the ADO II (Abbott, USA) device is far softer than previous devices, as it has no polyester fabric and can be easily delivered via an antegrade or retrograde approach through a 4 F or 5 F delivery catheters [11, 12]. Herein, we report our experience with transcatheter closure of pmVSD and icVSD using the ADO II in children.

## 2. Methods

Forty-one children, who underwent transcatheter closure of pmVSD and icVSD using the ADO II in Guangdong Provincial People's Hospital (Guangzhou, China) between March 2014 and June 2020, were enrolled in this study. All participants had isolated ventricular septal defect with a diameter < 5 mm. Most fulfilled at least one of the following the criteria: recurrent respiratory infections, failure to thrive, or significant hemodynamic compromise (including signs of left ventricular enlargement on electrocardiography (ECG), cardiomegaly on chest X-ray, or echocardiographic left atrial and/or left ventricular enlargement). The exclusion criteria were as follows: moderate-to-severe pulmonary hypertension, combined with other congenital heart defects requiring surgery, active local/systemic bacterial infections, VSD larger than 5 mm, body weight < 10 kg, and moderate-to-severe aortic regurgitation (AR).

All patients underwent comprehensive periprocedural transthoracic echocardiography (TTE). pmVSD and icVSD were defined as defects located at 9–12 o'clock and 12–1:30 positions, respectively, in the short axis parasternal view [13]. The subaortic rim (SAR) was measured from the upper margin of the defect to the aortic valve in the five-chamber view and parasternal long axis view. AR was classified as trivial (jet width/LVOT diameter < 10%), mild (jet width/LVOT diameter = 10%–24%), moderate (jet width/LVOT diameter = 25%–49%), or severe (jet width/LVOT diameter > 50%). Tricuspid regurgitation (TR) was classified as trivial (within 1 cm of the valve), mild (regurgitant jet area (RJA)/right atrial area (RAA) < 19%), moderate (RJA/RAA = 20%–40%), or severe (RJA/RAA > 41%) [14]. Residual shunt was assessed by the width of the color jet at the point of exit through the ventricular septum and classified as trivial (<1 mm color jet width), mild (1-2 mm color jet width), moderate (2-3 mm color jet width), or severe (>3 mm color jet width) [15].

Aortic valve prolapse (AVP) was graded into three degrees according to the morphology of the right coronary leaflet at the end of diastole during angiography: mild (buckling of the aortic cusp down the left ventricular outflow tract with minimal herniation into the VSD), moderate (prolapse of the cusp and its sinus with obvious herniation into the VSD), and severe (prolapse of the cusp and its sinus through the defect into the right ventricular outflow tract) [16, 17].

### 2.1. Device and Selection Protocol

The ADO II is a self-expanding fabric-free nitinol occluder consisting of dual symmetrical retention and flexible discs connected by a central waist. The occluders are available in two lengths (4 and 6 mm) and four waist diameters (3, 4, 5, and 6 mm). The retention discs have a diameter 6 mm greater than the waist size.

**Figure 1:**
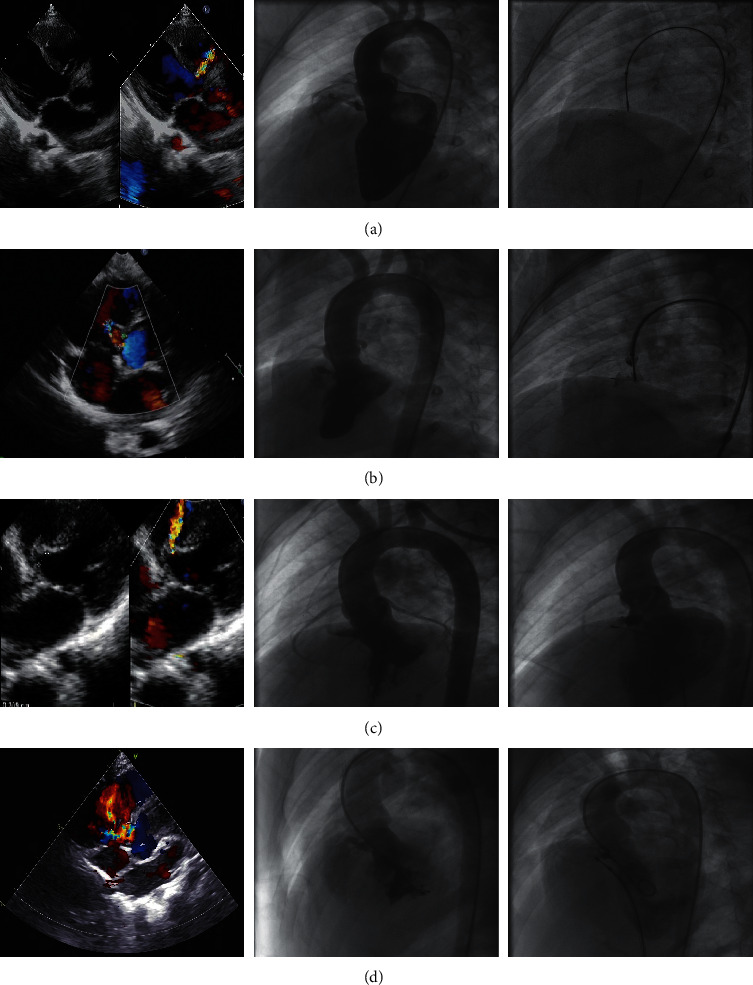
Transthoracic echocardiography (TTE) and angiographic findings considered suitable for ADO II implantation: (a) tubular aneurysm, (b) aneurysm with two constrictions, (c) aneurysm with an elongated conical appearance, and (d) aneurysm with multiple small exits.

For icVSD and pmVSD without a membranous aneurysm, the waist diameter selected was 1 to 2 mm larger than the VSD defect size in the case of sufficient SAR or 1 mm (±0.5 mm) larger for deficient SAR. For VSD associated with a membranous aneurysm, implantation of the ADO II was considered in selected cases with suitable anatomic configurations as illustrated in Figures 1(a)–1(d). In these cases, the disk diameter selected covered the entire entry, specifically 1 to 2 mm larger than the LV entry diameter for a sufficient SAR or equal to the LV entry diameter for a deficient SAR. A larger waist size was selected if sufficient SAR since elongation of the device through a relatively long duct decreases the effective diameter of the central waist. The device length (4 or 6 mm) was selected based on VSD depth as measured on angiography.

For pmVSD and icVSD associated with AVP, the defect size is often underestimated on TTE. Therefore, the effective LV entry diameter should be measured based on multiple TTE views. Furthermore, we considered the larger of the jet width measured on angiography or TTE images to aid in selecting a device waist size. The waist diameter selected was 1 mm (±0.5 mm) larger than this measurement. The device length selected was generally 4 mm.

### 2.2. Procedure

The procedure has been described in detail in previous publications. Briefly, standard right and left cardiac catheterization and left ventriculography and aortography (left anterior oblique 60°/cranial 20° projection for pmVSD and left anterior oblique 70°–80°/cranial 20° for icVSD) were performed in all cases. Two methods of device deployment were employed. The conventional technique involves an antegrade approach, with the formation of an arteriovenous loop, and initial deployment of the LV disc followed by RV disc. In the retrograde method, the delivery system is advanced over a long exchangeable wire through femoral artery without creating an arteriovenous loop, followed by the initial deployment of the RV disc and the LV disc thereafter. We prefer a tangential fluoroscopic projection (left anterior oblique 40°/cranial 20°) when releasing the device, as this provides a clear view of the relationship between the device and the ventricular septum. As the disc conforms to ventricular septum, it orients itself horizontally, and its position becomes relatively fixed. Subsequently, the waist and proximal disc can be released. In some cases, when appropriate disc conformance with ventricular septum cannot be confirmed, TTE is useful to verify the location and monitor whether the neighboring valve is affected.

### 2.3. Follow-Up

Patients without complications were discharged 24 hours after the procedure. All patients underwent chest radiography, electrocardiography, and TTE before discharge. Oral aspirin (5 mg/kg daily) was prescribed for 6 months. Follow-up visits were scheduled at 1, 3, and 6 months and annually thereafter. All visits included a routine physical examination, electrocardiography, and TTE. Serious complications relating to the procedure or device included (1) death, (2) Mobitz II atrioventricular block or complete atrioventricular heart block (CAVB), (3) new onset of more than moderate aortic regurgitation or tricuspid regurgitation, (4) tricuspid stenosis, (5) neurovascular events, (6) cardiac erosion, and (7) hemolysis.

### 2.4. Statistical Analysis

Continuous variables are expressed as median (range) and categorical variables as percentages and numbers of patients. Cumulative event-free survival was estimated using Kaplan–Meier analyses, and event-free survival curves were compared using the log-rank test. All analyses were performed using R 3.6.2 software.

## 3. Results

General procedural and follow-up characteristics of the 41 patients are summarized in Table 1. The baseline characteristics of the study population were not significantly different between pmVSD and icVSD. Implantation was successful in 40/41 patients (97.5%, 27/28 in pmVSD group, 13/13 in icVSD group). One patient with mild aortic valve prolapse in pmVSD group developed new-onset moderate aortic regurgitation after a 4/4 mm ADO II was deployed; however, this resolved after the device was retrieved and successfully replaced with a 5 mm zero eccentric VSD occluder. The antegrade approach was used in 33 patients (12 in icVSD group) and the retrograde approach in 8 patients (1 in icVSD group). A retrograde approach was selected in 3 patients due to technical difficulties and 1 patient due to a femoral venous malformation. For the remaining 4 patients, a retrograde approach was planned ahead of the procedure. In 1 patient, a retrograde approach resulted in device interference with the aortic valve resulting in moderate AR. On subsequent switching to an anterograde approach, the AR disappeared.

In the pmVSD group, 5 patients with mild AVP had trivial preoperative AR, among which the AR resolved postoperatively in 4 patients and remained unchanged in 1 patient after the procedure. New-onset trivial AR was observed in 4 patients with preoperative mild AVP. Among the 7 patients with mild preoperative TR, TR disappeared in 2, remained unchanged in 4, and progressed to moderate TR in 1 patient after the procedure. New-onset mild TR was observed in 6 patients. In the icVSD group, 1 patient presented with trivial preoperative AR that remained unchanged after the procedure. New-onset trivial AR was observed in 1 patient with preoperative mild AVP. New-onset mild TR was observed in 1 patient.

**Figure 2:**
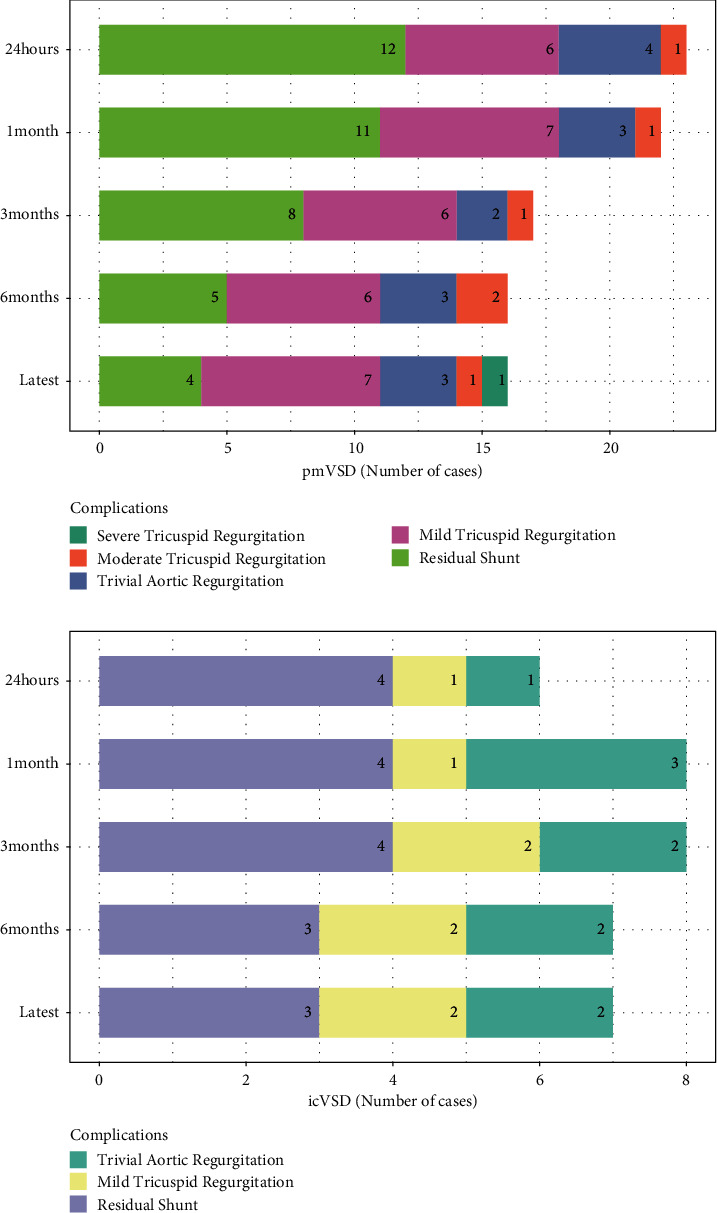
Progression of new-onset complications on follow-up.

**Figure 3:**
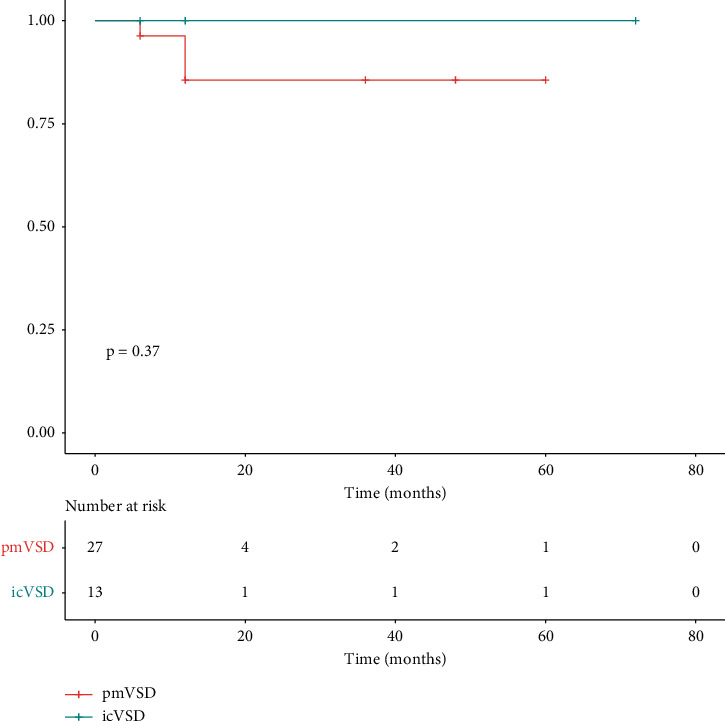
Kaplan–Meier curve depicting freedom from complications across follow-up.

Follow-up data were available for all patients. The median follow-up for the participants was six months (total range: 6 to 72 months).  Figure 2 shows the progression of new-onset complications on follow-up. One patient in pmVSD group with preexisting tricuspid regurgitation progressed to severe tricuspid regurgitation at the one-year follow-up. He was asymptomatic and continued to undergo close follow-up. One patient in the pmVSD group developed moderate tricuspid regurgitation at the 6-month follow-up. She was asymptomatic and her right atrium appeared normal on TTE. No deaths, AV block, moderate or worse aortic regurgitation, tricuspid stenosis, neurovascular events, cardiac erosion, or hemolysis occurred during the follow-up. Kaplan-Meier analyses (Figure 3) revealed no significant differences in the probability of complications between pmVSD and icVSD groups (*P*=0.37, log-rank test). No surgical or percutaneous reintervention was scheduled on this period of follow-up.

## 4. Discussion

This study demonstrates that transcatheter closure of pmVSD and icVSD less than 5 mm in diameter with the ADO II device is feasible and safe in children. This is also true for pmVSDs with a subaortic rim ≤ 2 mm or aortic valve prolapse.

### 4.1. Benefits of Utilizing ADO II in pmVSD

pmVSD close to the aortic valve (≤2 mm) commonly leads to aortic valve prolapse and subsequent aortic regurgitation because of the Venturi effect [18]. Typically, conventional symmetrical device implantation may worsen preexisting AR or result in new-onset AR.

We found that the ADO II is safe to implant in patients with a relatively deficient aortic rim (up to 2 mm). As the upper rim of the ADO II is 3 mm larger than the waist, a subaortic rim > 3 mm would generally be required to avoid aortic regurgitation [19]. However, we found that absent SAR or even more the presence of an AVP were not limitation factors. This is possibly because the soft structure of the device allows it to move freely with the aortic valve leaflet without disrupting the aortic valve motility when deployed in a VSD with a deficient aortic rim.

**Figure 4:**
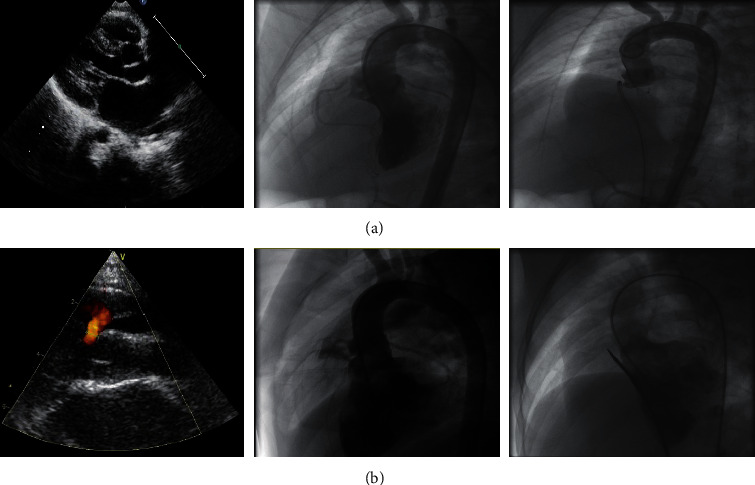
(a) Ventricular septal defect with severe aortic valve prolapse. The right coronary cusp prolapses into the right ventricular outflow tract (left, center). A 5/4 mm Amplatzer duct occluder II was successfully implanted without aortic regurgitation as shown by the angiography (right). (b) Intracristal ventricular septal defect. Transthoracic echocardiography (left) shows the defect is close to aortic valve. Left ventricular angiography at 70° left anterior oblique and 20° cranial projection was performed to visualize the defect (center). A 5/4 mm Amplatzer duct occluder II completely closed the defect completely without residual shunt or interfering with the aortic valve (right).

In our experience, implantation of ADO II in cases with severe AVP (*n* = 3) did not result in AR after the implantation (Figure 4(a)). ADO II was also found to be suitable for closure of pmVSD associated with a membranous aneurysm in selected cases with amenable morphologies. These morphologies are illustrated in Figures 1(a)–1(d). Generally, aneurysms with a small exit on the right side can be closed with the ADO II. In such cases, we recommend a retrograde approach because the delivery sheath is usually difficult to advance through an antegrade approach.

### 4.2. Benefits of Utilizing ADO II in icVSD

icVSD is located close to the aortic valve and is usually associated with aortic valve prolapse. With such defects, closure with the soft ADO II may be beneficial as it does not interfere with aortic valve function (Figure 4).

Other devices have also been used for the closure of icVSD. Qin et al. reported the zero eccentric VSD occluder can also be used to close icVSD with a successful closure rate of >90%. However, 2/38 patients developed AR, requiring surgical repair [3, 7]. In contrast, using the ADO II device for similar defects, we did not observe any AR requiring surgical repair.

Furthermore, Qin et al. observed considerably longer procedural and fluoroscopic times with the zero eccentric VSD occluder. Multiple procedure related issues may contribute to increased fluoroscopic time [20]. First, as the occluder is asymmetrical, it must be maneuvered back and forth to ensure the platinum marker on the left disk is positioned toward the apex. Additionally, the delivery sheath of the zero eccentricity VSD occluder is thicker (4-5 Fr versus 6–8 Fr.) and therefore less flexible than the ADO II. Therefore, it is difficult to maneuver into the left ventricle [21].

Overall, our observations suggest that the ADO II occluder is safer, easier to implant, and may require less fluoroscopic and procedural time than the zero eccentric VSD occluder system, particularly when delivered retrogradely avoiding the A-V circuit formation. However, further research comparing the two devices is required to confirm these speculations.

### 4.3. Antegrade versus Retrograde Approach

In most cases, the procedure was performed using the antegrade method in our study. This technique creates a stable line through which the delivery system can be advanced via venous access and avoids the risk of arterial injury in young children. In addition, the antegrade technique allows better control on the aortic disc positioning, and therefore, it, theoretically, should be the preferred technique for patients with deficient aortic rim and/or in VSD with AVP.

Initially, we reserved the retrograde approach for the following situations: (1) venous closure could not be performed due to technical difficulties, including guidewire entrapment within the chordal elements of the tricuspid valve and inability to advance the delivery sheath due to angulation of the path or a small defect; (2) bilateral femoral venous malformations. However, with accumulating experience, we have found that the retrograde approach involves fewer steps and reduces procedural costs (a snare set that costs more than 3000 yuan if not used). With this in mind, we recommend that the retrograde approach can be planned ahead of the procedure in select cases such as VSD with small exit.

### 4.4. Residual Shunts

Residual shunts were common in the immediate postoperative period. In the pmVSD group, complete closure was achieved in 55.5% at 24 hours and 85.2% at the latest follow-up, respectively. In the icVSD group, complete closure was achieved in 69.2% at 24 hours and 76.9% at the latest follow-up, respectively. However, these residual shunts were not hemodynamically significant as the heart murmur disappeared or decreased in intensity (grade 1–2/6) in all children. Further, no hemolysis or endocarditis was observed in our sample.

Other studies utilizing the ADO II for VSD occlusion also observed a similar trend. For instance, Wang et al. reported 32/45 (71.1%) trivial-to-mild residual shunts immediately after transcatheter closure of outlet-type VSDs with the ADO II device, which gradually reduced to 19/45 (42.2%) at the latest follow-up (range: 0.3–51.1 months) [22]. Lyu et al. reported 10/51 (19.6%) instances of trivial residual shunts after transcatheter closure of perimembranous VSD using ADO II and a 100% complete closure rate by the six-month follow-up [23].

We suspect that the high incidence of early shunts can be attributed to the soft fabric-free design of the ADO II. However, eventual thrombosis and occluder endothelialization may contribute toward residual shunt resolution [24]. Given the high rate of resolution for early shunts associated with ADO II implantation, a higher level of early shunting is acceptable during implantation. In our experience, observing a reduction or resolution of the heart murmur and confirming stable device position and optimal conformation on fluoroscopic imaging are sufficient to release the device, even if residual shunts are visible on TTE. However, some residual shunts may persist. This may be due to device-defect mismatch or a failure to close the LV entry. In our sample, 7 cases had residual shunts on the latest follow-up. However, none of them had a heart murmur suggesting that the residual shunt may not be hemodynamically significant. Furthermore, none of our patients developed endocarditis or hemolysis during follow-up. Several reasons may cause a device-defect mismatch, such as an underestimation of defect size, or apprehension to implant an oversized device that may cause damage to the aortic valve. Therefore, we should aim to completely close the LV entry and device selection should be made according to LV entry diameter.

### 4.5. Limitations

This study is limited as it is a retrospective study. Furthermore, interobserver variability may have influenced the findings as postoperative TTE and outpatient TTE on follow-up were performed by different pediatric cardiologists. Additionally, only a small sample size was reported since the use of ADO II was reserved to close challenging VSD as its high cost limits its widespread clinical use in developing countries. Finally, long-term outcomes cannot be determined because the follow-up period was relatively short.

## 5. Conclusion

The ADO II provides a feasible and safe alternative for the closure of perimembranous and intracristal ventricular septal defects with a diameter less than 5 mm in children. Yet, efforts should be made to entirely close the LV entry aiming to reduce the unacceptable rate of residual shunt in order to improve its efficacy.

## Figures and Tables

**Table 1:** Baseline and procedural characteristics of the study sample.

	(All)	pmVSD group	icVSD group	*P* value
	*N* = 41	*N* = 28	*N* = 13	
Age (years)	3.5 [0.9–12.0]	3.6 [1.8–12.0]	3.3 [0.9–12.0]	0.933
Gender				0.987
** **Male	22 (53.7%)	15 (53.6%)	7 (53.8%)	
** **Female	19 (46.3%)	13 (46.4%)	6 (46.2%)	
Weight (kg)	15.0 [10.0–43.0]	15.2 [11.0–29.5]	15.0 [10.0–43.0]	0.674
Height (cm)	98.0 [73.0–152.0]	97.5 [80.0–141.0]	98.0 [73.0–152.0]	0.758
Aortic regurgitation				0.391
** **None	35 (85.3%)	23 (82.1%)	12 (92.3%)	
** **Trivial	6 (14.7%)	5 (17.9%)	1 (7.7%)	
Tricuspid regurgitation				0.698
** **None	30 (73.2%)	21 (75.0%)	9 (69.2%)	
** **Mild	11 (26.8%)	7 (25.0%)	4 (30.8%)	
AVP				0.031
** **None	8 (19.5%)	9 (32.1%)	0 (0.00%)	
** **Mild	30 (73.2%)	16 (57.1%)	13 (100%)	
** **Severe	3 (7.3%)	3 (10.7%)	0 (0.00%)	
SAR				0.090
** **≤2 (mm)	31 (75.6%)	19 (67.9%)	12 (92.3%)	
** **>2 (mm)	10 (24.4%)	9 (32.1%)	1 (7.69%)	
Systolic PAP (mmHg)	28.0 [14.0–38.0]	28.0 [14.0–38.0]	28.0 [20.0–37.0]	0.683
Diastolic PAP (mmHg)	10.0 [3.0–17.0]	10.0 [3.0–16.0]	10.0 [4.0–17.0]	0.810
Mean PAP (mmHg)	16.0 [6.0–22.0]	16.0 [6.0–22.0]	16.0 [10.0–22.0]	0.725
Qp/Qs	1.36 [1.1–2.0]	1.33 [1.1–2.0]	1.47 [1.1–1.9]	0.518
Vascular approach				0.193
** **Antegrade	33 (80.5%)	21 (75.0%)	12 (92.3%)	
** **Retrograde	8 (19.5%)	7 (25.0%)	1 (7.69%)	
Procedure time (min)	64.0 [55.0–78.0]	63.5 [55.0–77.0]	67.0 [64.0–79.0]	0.501
Immediate RS				0.605
** **None	24 (60.0%)	15 (55.6%)	9 (69.2%)	
** **Trivial	4 (10.0%)	2 (7.41%)	2 (15.4%)	
** **Mild	8 (20.0%)	7 (25.9%)	1 (7.69%)	
** **Moderate	4 (10.0%)	3 (11.1%)	1 (7.69%)	
RS at the latest follow-up				0.215
** **None	33 (82.5%)	23 (85.2%)	10 (76.9%)	
** **Trivial	2 (5.00%)	0 (0.00%)	2 (15.4%)	
** **Mild	4 (10.0%)	3 (11.1%)	1 (7.69%)	
** **Moderate	1 (2.50%)	1 (3.70%)	0 (0.00%)	

VSD: ventricular septal defect; pmVSD: perimembranous VSD; icVSD: intracristal VSD; AVP: aortic valve prolapse; SAR: subaortic rim; PAP: pulmonary arterial pressure; *Q*_*p*_/*Q*_*s*_: pulmonary to systemic blood flow; RS: residual shunt.

## Data Availability

The data used to support the findings of this study are available from the corresponding author upon request.
